# Delayed mirror visual feedback presented using a novel mirror therapy system enhances cortical activation in healthy adults

**DOI:** 10.1186/s12984-015-0053-1

**Published:** 2015-07-11

**Authors:** Hsin-Min Lee, Ping-Chia Li, Shih-Chen Fan

**Affiliations:** Department of Physical Therapy, I-Shou University, Kaohsiung, Taiwan Republic of China; Department of Occupational Therapy, I-Shou University, Kaohsiung, Taiwan Republic of China

**Keywords:** Digital mirror therapy, Event-related desynchronization, Mirror neuron system, Mirror visual feedback, Movement training, Mu rhythm EEG

## Abstract

**Background:**

Mirror visual feedback (MVF) generated in mirror therapy (MT) with a physical mirror promotes the recovery of hemiparetic limbs in patients with stroke, but is limited in that it cannot provide an asymmetric mode for bimanual coordination training. Here, we developed a novel MT system that can manipulate the MVF to resolve this issue. The aims of this pilot study were to examine the feasibility of delayed MVF on MT and to establish its effects on cortical activation in order to understand how it can be used for clinical applications in the future.

**Methods:**

Three conditions (no MVF, MVF, and 2-s delayed MVF) presented via our digital MT system were evaluated for their time-course effects on cortical activity by event-related desynchronization (ERD) of mu rhythm electroencephalography (EEG) during button presses in 18 healthy adults. Phasic ERD areas, defined as the areas of the relative ERD curve that were below the reference level and within -2–0 s (P0), 0–2 s (P1), and 2–4 s (P2) of the button press, were used.

**Results:**

The overall (P0 to P2) and phasic ERD areas were higher when MVF was provided compared to when MVF was not provided for all EEG channels (C3, Cz, and C4). Phasic ERD areas in the P2 phase only increased during the delayed-MVF condition. Significant enhancement of cortical activation in the mirror neuron system and an increase in attention to the unseen limb may play major roles in the response to MVF during MT. In comparison to the no MVF condition, the higher phasic ERD areas that were observed during the P1 phase in the delayed-MVF condition indicate that the image of the still hand may have enhanced the cortical activation that occurred in response to the button press.

**Conclusions:**

This study is the first to achieve delayed MVF for upper-limb MT. Our approach confirms previous findings regarding the effects of MVF on cortical activation and contributes additional evidence supporting the use of this method in the future for upper-limb motor training in patients with stroke.

**Electronic supplementary material:**

The online version of this article (doi:10.1186/s12984-015-0053-1) contains supplementary material, which is available to authorized users.

## Background

Since Dr. Ramachandran first introduced mirror therapy (MT) to relieve the phantom pain experienced in the limbs of amputees in 1992 [1], MT has been used to manage many other conditions related to pain and motor disorders [2–4]. Among them, the applications of MT for patients with hemiplegic arms after stroke have drawn much attention in the past two decades [5–7]. In the conventional clinical setup, a plain mirror or mirror box is used to reflect the active hand to form an illusory hand, which is visually superimposed on the impaired hand. The patients are asked to turn their head to observe the illusory hand in the mirror and to persuade themselves that the impaired hand can move as well as the active hand at that moment [8]. Similar to action observation (AO) [9, 10], the impact of the visual stimulus (mirror visual feedback [MVF]) on the patient’s brain is thought to cause cortical reorganization, therefore enhancing motor recovery of the paretic limbs [6, 11]. As a motor rehabilitation approach for hemiparetic arms, traditional MT is advantageous because it is inexpensive, easy to setup, and convenient to use. However, traditional MT has its limits in the training mode in terms of bimanual coordination movements, i.e., MT can only train symmetric movements, not reciprocal or alternating movements [12], as the illusory hand always reversely synchronizes with the active hand. Previous studies have shown that patients with stroke had impaired gross and fine motor coordination during bimanual movement (in both symmetric and asymmetric modes) [13, 14]. It was also found that bilateral arm training with symmetric and asymmetric movement patterns can improve both the interlimb and intralimb coordination of patients with stroke [13]. Regarding the benefits of bimanual coordination training in clinics, it is important to determine whether the MVF (illusory hand) can be manipulated (e.g., delayed) by digital technology to create a novel application of MT for bimanual motor rehabilitation of paretic limbs in patients with stroke.

To the best of our knowledge, only a few studies have tried to extend the concepts of MT to create a new interface for MT training. For example, one study utilized a video-optic system to play prerecorded movements of the active hand on a tilted mirror placed in front of the subjects [15]. The subjects placed their impaired hand just under the illusory hand and tried to train the brain to re-establish the cortical representation of that hand. Two of the three subjects with phantom pain from brachial plexus avulsion were able to relieve their pain successfully with this method, and the corresponding cortical activities detected by functional magnetic resonance imaging (fMRI) increased in these two subjects. In addition, two different virtual reality (VR)-based platforms that used a computer-graphic (CG) virtual hand to represent the illusory hand have been employed to treat phantom pain [16] or complex regional pain syndrome [17] in an immersive or non-immersive way, respectively. The major limitation of these previous MT-like technologies may be the sense of reality for the illusory hand during motor training. The lower-resolution MVF images (640 × 480 pixels) of the illusory hand [15] or CG hand on the head-mounted display [16] or front screen [17] used in these earlier studies may not have been real enough to persuade the subject in a first-person perspective sense. Furthermore, to our knowledge, no studies have used video or VR technologies to manipulate the MVF to accomplish different bimanual coordination movements.

Studies using fMRI and transcranial magnetic stimulation (TMS) have shown that MVF of the active hand in MT can instantly increase neuronal activity in the region of the sensorimotor cortex that represents the non-active hand in normal subjects [18, 19] and in patients with stroke [11, 20]. These findings indicate that MVF can affect the brain via certain perceptuo-motor control processes, which are also essential for regular motor training approaches in terms of motor learning principles [21]. However, the fMRI studies mainly aimed to find the areas of the brain that corresponded to the MVF stimulus, while the TMS studies have technical constraints, as they cannot reveal continuous changes in cortical activity. Therefore, the instant time-course changes in cortical areas responsive to MVF are currently unclear. As a time-course study of cortical activation during MT may help determine when and how the brain responds to MVF, it is important to find a suitable tool for examining such temporal patterns of brain activity during MT. It is also essential to understand the possible therapeutic effects of MVF on the lesioned brain.

The mu rhythm is a specific frequency range (8–12 Hz) in the electroencephalography (EEG) signal that is recorded over the sensorimotor cortical areas. The amplitude decrease in mu-rhythm power (called event-related desynchronization [ERD]) can be used to depict the temporal pattern of cortical activity when preparing, producing, and controlling movement events [22, 23]. Mu-rhythm ERD has been used in previous studies to evaluate and interpret the effects of MT for patients with stroke [24] and has revealed lower cortical neuronal network activity in both the lesioned and intact hemispheres of patients stroke [25]. Previous studies also suggest that mu-rhythm ERD during AO may be an indicator of activity in the mirror neuron system (MNS) [26], which is hypothesized to be the potential neural network underlying the mechanisms of MT [6]. Therefore, measuring the mu-rhythm ERD during MT training should be an ideal method for revealing the time-course changes in cortical activity in response to an MVF stimulus.

In this study, we hypothesized that the MVF effects on cerebral activation would be enhanced with a new form of MT interface that has two distinct features: (1) vivid MVF with a high-resolution (1920 × 1080 pixels) and first-person perspective view for clinical motor training based on MT concepts, and (2) time-delayed MVF to achieve coordination training for the wrist and hand. Therefore, the aims of this pilot trial were to assess the feasibility of this novel MT system for activating cortical areas in healthy adults and to compare the conditions of instant and delayed MVF with the condition of no MVF. We used the decrease in mu-rhythm power (ERD) to determine the temporal patterns of these three test conditions, which were presented with our MT system. The potential underlying mechanisms of MT are also discussed.

## Methods

### Digital MT system

**Fig. 1:**
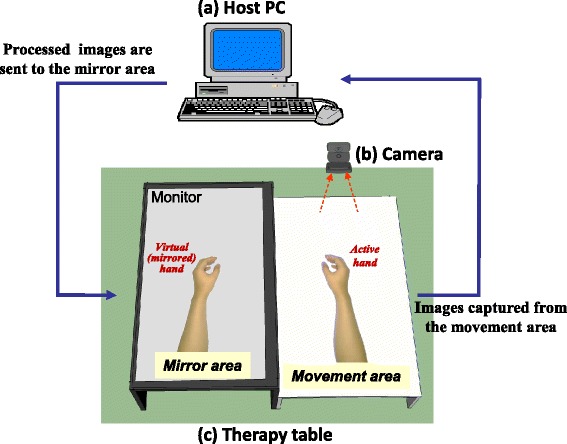
Diagram of the digital mirror therapy system. The host personal computer (PC) (**a**) captures images of the active hand via a high definition (HD) webcam (**b**) placed above the movement area of the therapy table (**c**). The host PC sends the processed images (vertically mirrored) to the mirror area (an HD monitor) of the therapy table

As shown in Fig. 1, the digital MT system consists of a host personal computer (PC), camera, and therapy table with a movement area and a mirror area. The participant sits behind the table and places their non-impaired hand (active hand) on the movement area. In the mirror area of the table, a slim 27-in. liquid crystal display (LCD) monitor with a resolution of 1920 × 1080 pixels (Model IPS277L-BN; LG Inc.) was situated slightly above the table to reserve the underlying space for the non-active hand. Continuous images of the movement area were recorded with a video camera (C920, resolution: 1920 × 1080 pixels; Logitech Inc.) positioned above the area at a frame rate of 30 frames per second and were sent to the host PC, which processed the vertically mirrored and/or time-delayed images. A custom-made LabVIEW program (Ver. 2012, National Instruments Corporation) for the PC was used to process the acquired images and continuously send them to the monitor on the mirror area of the table to represent the mirrored images of the active hand’s movement. Before the experiment, a reference object such as a computer mouse was used to calibrate the camera orientation by adjusting the camera tripod to correspond to the movement and mirror areas.

### Subjects

Twenty-eight healthy adults volunteered to participate in the study. After confirming the presence of obvious mu waves in our testing environment, 18 healthy young adults (11 men and 7 women, mean age = 20.9 years, standard deviation [SD] = 1.7) were chosen to participate in the following experiments to test our hypothesis. All participants were right-handed and had normal or corrected-to-normal vision. The experimental procedures were approved by the local Human Research Ethics Committee. After receiving a general description of the experiment, all recruited subjects provided written informed consent before participating in the study.

### Experimental paradigm

All of the experiments were conducted in the same bright room with air conditioning to keep the temperature as stable as possible. Participants were seated in a height-adjustable chair with their forearms resting on the therapy table. They were asked to repeatedly press a square button with their right index and middle fingers in a self-paced way. Since the time factors (press duration and interval) might affect the recovery time of mu-rhythm suppression [27], the self-paced press movement was trained for several minutes to keep the press duration below 0.5 s and the interval between 15 and 20 s. During the experiment, subjects were instructed to relax and rest their index and middle fingers on the button for the entire duration of the experiment. Subjects were asked not to move any other body parts to the greatest extent possible and to keep their eyes on the central mirror area. Eye blinking was not allowed during the button press. A video camera (C930e; Logitech Inc.) placed in front of the subject was used to monitor compliance. To prevent the subject from being distracted during the test and to enhance the test response [28], an opaque plastic divider was placed vertically in the midsagittal plane of the table to keep the subject from seeing the active hand.

Three different visual feedback conditions were used to test their impact on cortical activity. In the first condition, the mirror area showed a still image of the movement area without the hand (no-MVF condition). In the second condition, the mirror area showed simultaneous mirrored images of the movements of the right hand (MVF condition). In the third condition, mirrored images of the movements of the right hand were shown in the mirror area with a 2-s delay (delayed-MVF condition). To enhance the feeling of the mirrored illusion, the left hand rested on a platform with an identical button on it and we asked the subjects to keep their left hands relaxed during the experimental conditions. To reduce order effects, the three conditions were randomly arranged for each subject. In each condition, the subject pressed the button in the self-paced manner described above until they had completed at least 50 repetitions, which were counted by the experiment assistant. Subjects were allowed to rest for 5 min between conditions. To reduce effects of the previous condition and obtain a more stable mu-rhythm energy level, a 1-min eyes-closed period was included before the start of the button presses in each condition. The entire experiment lasted 2 to 3 h, which included setup time, button press practice, and all three experimental conditions.

### Force and surface electromyography (sEMG) measurements

The custom-made square button (7 × 4 × 0.6 cm) was fixed on the centre of the movement area and a miniature load cell (LBS-50 lb; Interface Inc.) placed beneath the button recorded the press force during the experiment. The force signal was also used to confirm in real time that the button presses were occurring with the durations and intervals we requested. To provide a reference point for the start of the movement (start of button press) [29], a pair of surface electrodes (Medi-Trace 200; Kendall Inc.) was placed on the finger flexors of the active hand to record the sEMG activity. The electrodes were placed in parallel on the corresponding muscle belly with a 4-cm inter-disc distance, and the attachment location was decided by palpation of the active muscle area when the subjects were asked to flex their index and middle fingers. The finger extensor of the active hand was also monitored to confirm relaxation of the forearm when the subject was not pressing the button. The sEMG signals were amplified (gain: ×5000) and band-pass filtered (30–1000 Hz) with an amplifier (Grass QP511; Astro-Med Inc.). The finger flexors of the non-active hand were also monitored in the same way to confirm relaxation of the left hand during the experiment.

### EEG measurements

EEG recordings were obtained from three Ag/AgCl electrode pairs (F-E5GH; Grass-Telefactor Inc.) placed around the standard C3, C4, and Cz scalp sites based on the International 10–20 system. The Cz, C3, and C4 electrode pairs (inter-electrode distance: 2.5 cm) covered the supplementary motor area (SMA), and the contralateral (active hand) and ipsilateral (non-active/mirrored hand) primary sensorimotor hand areas, respectively. The electrode impedance for all electrodes was checked using an impedance meter (EIM-107 Prep-Check Plus; General Devices Inc.) and was kept below 5 kΩ. The reference electrode was placed on the right ear lobe. To prevent contamination of the recordings from ocular artefacts, the vertical electrooculogram (EOG) was also monitored with a pair of electrodes (Medi-Trace 100; Kendall Inc.) placed above and below the left eye. The recorded EEG signals were sent to the amplifier (Model QP511; Astro-Med, Inc.) with a gain of 20,000 and a band-pass filtering of 0.1 ~ 100 Hz. All of the EEG, EOG, force, and sEMG signals were finally sent to the PC for further analysis via a data acquisition device (PCI-6259; National Instruments Inc.) with a sampling rate of 1000 Hz. The experimenter was also able to monitor the data on-line using a monitor to confirm the quality, thus allowing additional presses to be requested if any press movements failed.

### ERD variables

**Fig. 2:**
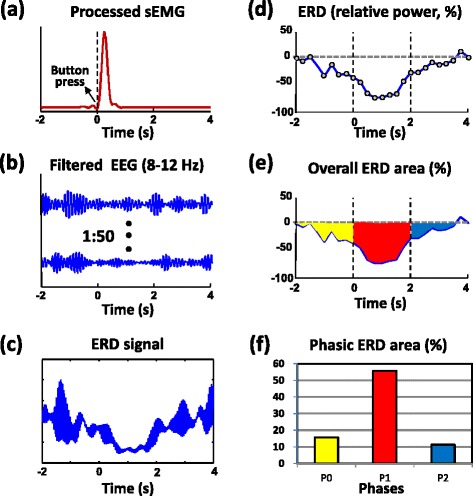
Processing of the event-related desynchronization (ERD) curve, overall ERD area, and phasic ERD areas (data from the Cz channel of one subject in the no-mirror visual feedback condition). **a** The processed surface electromyography (EMG) signal was used to define the start point of the press movement. **b** Filtered electroencephalography (EEG) epochs (*n* = 50) for the mu rhythm (8–12 Hz). **c** The filtered EEG epochs were squared and averaged to form the mu rhythm ERD signal. **d** The relative ERD power curve ( % of the reference level) was formed by smoothing the raw ERD signal (each grey dot over the 250-ms time window). **e** The overall ERD area was defined as the averaged area of the entire ERD curve below the reference level. We used the area to quantify the cortical activation in response to the button press. **f** According to the phasic ERD areas for the P0 (-2–0 s), P1 (0–2 s), and P2 (2–4 s) phases (yellow, red, and blue areas in (**e**), respectively), a time-course change in cortical activation can be observed

To retrieve the mu-rhythm ERD epochs from the EEG signal, the sEMG signal recorded from the active hand was sequentially processed with band-pass filtering (30 ~ 300 Hz), band-reject filtering (60, 120, and 180 Hz), rectification, and low-pass filtering (3 Hz). By visually marking the point at which the processed EMG signal increased (considered as the 0-s point of each press) [29], EEG epochs of -2 to 4 s were extracted and further analysed for each EEG channel (as shown in Fig. 2a and b). We selected 8–12 Hz as the frequency band in which mu waves occurred and followed the standard procedures of ERD processing suggested by Pfurtscheller and Lopes da Silva [30]. The intertrial variance method was used to remove the possible contribution of phase-locked event-related potentials [31]. Briefly, a total of 50 raw EEG epochs were band-pass filtered (8–12 Hz), had their mean trend removed, were squared for each data point, and finally averaged across all epochs to obtain a mu-rhythm power amplitude signal (Fig. 2b and c). Furthermore, the relative power of the ERD waveform was calculated as follows:

1 $$ \mathrm{E}\mathrm{R}\mathrm{D}\ \left(\%\right) = \left(\mathrm{A} - \mathrm{R}\right)/\mathrm{R} \times 100 $$

where *A* and *R* represent the mu-rhythm power amplitude and the reference level, respectively. A previous study showed that the mu ERD starts around 2 s prior to the onset of a voluntary self-paced finger movement and suggested that the power amplitude around 2 s should be used as the reference level [31]. In the current study, most subjects exhibited a higher mu-rhythm power amplitude at 1.8 to 2.2 s before the press movement in the no-MVF condition, thus we choose the averaged power amplitude at around -2 s (-2.125 to -1.825 s of the EEG epoch) of the no-MVF condition as the reference *R* for all conditions. To smooth the data and reduce variability [30], time averaging with a 250-ms window was also adopted (Fig. 2d).

To quantify the cortical activation during the -2 – 4 s surrounding the press movement, we used the averaged area of the entire ERD curve under the reference level as the amplitude parameter of cerebral activation. Further, we separated the entire ERD curve into three time phases: P0 (-2–0 s), P1 (0–2 s), and P2 (2–4 s). For each phase, the averaged ERD area was used to reveal the phasic amplitude of cerebral activation and to observe the time-course changes during the button press (Fig. 2e and f).

To characterize the lateralization of the MVF effects, a lateralization index (LI) was used:

2 $$ \mathrm{L}\mathrm{I} = \left({\mathrm{ERD}}_{\mathrm{R}} - {\mathrm{ERD}}_{\mathrm{L}}\right)/\left({\mathrm{ERD}}_{\mathrm{L}} + {\mathrm{ERD}}_{\mathrm{R}}\right) $$

where *ERD*_*R*_ and *ERD*_*L*_ represent the overall ERD areas (cerebral activation) of the C4 and C3 channels, respectively. An LI of 1 indicates total dominance of the non-active (mirrored) cortex and an LI of -1 indicates total dominance of the active cortex. Off-line processing of the force, EMG, EEG, and ERD data was completed by a custom-made MATLAB program (ver. 7.0; The MathWorks Inc.).

### Statistical analysis

One-way repeated measures analyses of variance (ANOVAs) of the press interval, press amplitude, and overall ERD area were used for each EEG channel to examine the main effects of the test conditions. Pairwise comparisons were performed to compare the differences between each pair of test conditions. Two-way repeated measures ANOVAs were used for each EEG channel to examine the main effects of the test conditions and time phases. Pairwise comparisons were then performed to compare the differences between each pair of test conditions for each time phase to determine the effect of the MVF and delayed MVF conditions in terms of the time course. A one-way repeated measures ANOVA for the lateralization index was used to test the main effect of the test conditions on activity in the mirror cortex. Pairwise comparisons were also performed to compare the differences between each pair of test conditions to observe the effects of the MVF and delayed MVF conditions. All variables were tested for normality using Mauchly’s test. When the assumption of sphericity was violated, significance was adjusted using the Greenhouse-Geisser correction. All statistical analyses were performed using SPSS (ver. 18.0; SPSS Inc.). Probability values of *P* < 0.05 were considered indicative of statistically significant results.

## Results

The averaged time interval and force amplitude of the button press for the three test conditions ranged from 17.8 to 19.0 s and from 22.1 to 22.6 N, respectively. One-way ANOVAs for the main effect of test condition on the time interval and force amplitude did not show statistical significance (time: F_2, 51_ = 0.41, *P* = 0.67; force: F_2, 51_ = 0.02, *P* = 0.98). Additionally, pairwise comparisons indicated that the time interval and amplitude of the button presses were not significantly different between the test conditions (P > 0.05 for all comparisons) (Table 1).

**Table 1:** Means (with standard deviations) of the averaged intervals and amplitudes of button presses for the three test conditions

(*n* = 18)	No-MVF	MVF	Delayed-MVF	*P* value
Interval (s)	18.4	17.8	19.0	0.57^a^, 0.11^b^, 0.51^c^
	(4.5)	(3.1)	(3.4)	
Amplitude (N)	22.1	22.6	22.0	0.71^a^, 0.64^b^, 0.99^c^
	(9.2)	(11.8)	(9.9)	

^a^No-MVF vs. MVF, ^b^MVF vs. Delayed-MVF, ^c^Delayed-MVF vs. No-MVF. MVF: mirror visual feedback

**Fig. 3:**
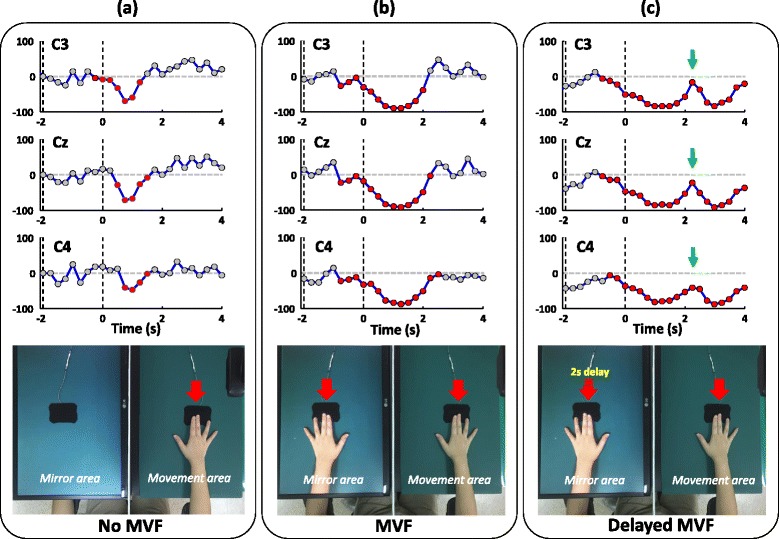
Typical examples of the time-course event-related desynchronization (ERD) waveforms of the C3, Cz, and C4 channels for all three test conditions: no-mirror visual feedback (MVF) (**a**), MVF (**b**), and delayed-MVF (**c**) conditions. The red circles mark the points where the mu power amplitude decreased from or increased to the relative peak and was lower than the reference level in response to the button press or MVF. **a** ERD mainly occurred after the button press. **b** ERD occurred earlier and with significantly larger amplitude in response to MVF compared to that observed with no MVF. **c** ERD reappeared when delayed MVF was provided around 2 s after the button press

Typical examples of the time-course changes of the ERD waveforms during the button presses for the three test conditions are shown in Fig. 3. In channel C4 of the no-MVF condition (Fig. 3a), a decrease in mu power (ERD) was noted after the button press and the power amplitude quickly recovered to the reference level (prior to 2 s). When MVF was provided (Fig. 3b), the decrease in mu power occurred much earlier, and was larger and longer than that during the no-MVF condition. Interestingly, when MVF was delayed for 2 s, the ERD waveform prior to 2 s was similar to that for the regular MVF condition, but a second decrease in mu power was noted 2 s after the button press (green arrows in Fig. 3c). For the other EEG channels, similar time-course changes were observed (Fig. 3).

**Fig. 4:**
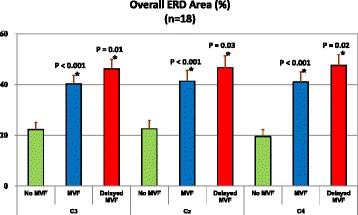
Averaged overall event-related desynchronization (ERD) areas (*n* = 18) with error bars (standard error) for the three test conditions (no mirror visual feedback [MVF]: green; MVF: blue, and delayed MVF: red) for the C3, Cz, and C4 channels, respectively. *Overall ERD areas of this test condition are significantly higher than the areas of the previous test condition for the same EEG channel. The overall ERD areas of the delayed-MVF condition are obviously larger than the areas for the no-MVF condition for all channels (all *P* < 0.001, not shown in the figure)

One-way ANOVAs of the main effect of test condition for the overall ERD areas during the period of -2–4 s showed statistical significance for all three EEG channels (C3: F_2, 51_ = 13.97; Cz: F_2, 51_ = 9.46; C4: F_2, 51_ = 15.85; all *P* < 0.001). As shown in Fig. 4, the ERD areas (%) in the delayed-MVF condition (C3: 46.2 ± 15.9; Cz: 46.6 ± 19.9; C4: 47.6 ± 17.9) were significantly larger than the areas in the MVF (C3: 40.3 ± 14.6; Cz: 41.4 ± 17.8; C4: 41.0 ± 16.8) and no-MVF (C3: 22.3 ± 11.5; Cz: 22.6 ± 14.0; C4: 19.5 ± 11.5) conditions for all channels (*P* < 0.05 for all comparisons). The ERD areas in the MVF condition were significantly larger than the areas in the no-MVF condition for all channels (*P* < 0.001 for all comparisons).

Furthermore, two-way ANOVAs of the phasic ERD areas showed that both the test condition and the time phase were significant factors for all three EEG channels (F values ranged from 23.02 to 47.81, all *P* < 0.001). Additionally, a significant interaction between the main factors was found (F values ranged from 8.76 to 14.30, all *P* < 0.01). As shown in Table 2, the phasic ERD areas of the P0, P1, and P2 phases in the MVF condition were significantly higher than the areas in the no-MVF condition (*P* < 0.05 for all comparisons) for all channels. However, only the phasic ERD areas of the P2 phase (2–4 s) in the delayed-MVF condition were significantly higher than the areas in the MVF condition (*P* < 0.05 for all comparisons).

**Table 2:** Means (with standard deviations) of the phasic ERD areas ( %) at each measured channel during the P0 (-2–0 s), P1 (0–2 s), and P2 (2–4 s) phases for the three test conditions

(*n* = 18)	No-MVF			MVF			Delayed-MVF		
Phases	P0	P1	P2	P0	P1	P2	P0	P1	P2
C3 channel	16.1	42.2	8.6	34.0^a^	61.7^a^	25.1^a^	35.5	59.7	43.4^a^
	(12.3)	(16.0)	(11.4)	(18.6)	(16.5)	(18.6)	(17.6)	(18.8)	(20.0)
Cz channel	12.0	43.4	12.3	29.0^a^	62.6^a^	32.5^a^	30.8	60.1	49.0^a^
	(11.7)	(20.6)	(15.7)	(18.2)	(19.5)	(21.1)	(19.5)	(19.1)	(25.8)
C4 channel	10.5	38.6	9.5	26.9^a^	63.1^a^	32.9^a^	30.8	59.6	52.4^a^
	(8.6)	(19.2)	(14.6)	(19.9)	(17.6)	(21.4)	(20.3)	(17.2)	(22.6)

^a^The phasic ERD areas are significantly higher than the areas in the same phase compared to the test condition to the left (*P* < 0.05) for each channel. ERD event-related desynchronization, MVF mirror visual feedback

Table 3 shows the averaged LIs with standard deviations for all three conditions. A one-way ANOVA of the main effect of condition for the LIs of the overall ERD area did not show statistical significance (F_2, 51_ = 1.88, *P* = 0.16). Pairwise comparisons showed that the LIs in the MVF and delayed-MVF conditions were significantly higher than the LI in the no-MVF condition (*P* < 0.05), although large variability was noted. No significant difference was found between the LIs of the MVF and delayed-MVF conditions (*P* = 0.3).

**Table 3:** Means (with standard deviations) of the lateralization index during the button presses for all test conditions

(*n* = 18)	No-MVF	MVF	Delayed-MVF	*P* value
Lateralization index	-0.09	-0.01	0.01	0.002^a^, 0.30^b^, <0.001^c^
	(0.18)	(0.13)	(0.18)	

^a^No-MVF vs. MVF, ^b^MVF vs. Delayed-MVF, ^c^Delayed-MVF vs. No-MVF. MVF: mirror visual feedback

## Discussion

In this study, a novel MT training system based on digital technology was developed to determine the effects of MVF on cortical activation. Rather than using the traditional physical mirror, the MVF in the current study involved processed movement images (30 frames per second) that were captured by a high-resolution camera and displayed on a high-resolution LCD screen (Figs. 1 and 3). Compared to traditional MT, the interface and setup of our MT training system for the upper limbs were changed in various ways, including an improved viewing angle for the MVF that produced less tension in the cervical posture of the subjects. Moreover, the superimposed MVF just above the real hand provided a better sense of body ownership for the virtual hand and may make the MVF more realistic compared to the MVF provided in previous studies with a head-mounted display [16] or front screen [17]. In terms of using MT as a specific form of bimanual training [6, 32], our system has the capacity to manipulate and delay the MVF to test the feasibility of bimanual coordination training in the future (see video, Additional file 1). To our knowledge, only one recent study has used a similar idea to manipulate MVF of the lower limbs for gait training under a VR-based environment [33]. Delayed MVF appears to not follow the underlying principle of traditional MT, which is to promote the coupling of two hands in a synchronous way [32]. However, it might work to stimulate brain circuits [12] other than those used for symmetrical bimanual movements in stroke patients with severe hand paresis.

In the present study, we primarily examined the effects of instant MVF and delayed MVF on central cortical activation using the power change in mu rhythm (i.e., ERD). With standardization of the press interval and amplitude for 18 healthy subjects (Table 1), we found that cortical activation (represented as the overall ERD areas) in the SMA and bilateral primary sensorimotor hand areas were larger when MVF was provided (Figs. 3 and 4). Furthermore, with the delayed MVF, cortical activation was reinitiated and prolonged (Fig. 3c). Time-course analysis also showed significant enhancement of cortical activity during all three phases (P0, P1, and P2) when MVF was provided, as shown in Table 2 (all channels). Especially, compared to the no-MVF and regular MVF conditions, the 2-s delayed MVF condition significantly enhanced cortical activation in the P2 phase (2–4 s). As the connection between mu rhythms and mirror neuron activity has been suggested by many previous studies (summarized in a review article [26]), our findings support the notion that the MNS plays a role in the cortical responses to MVF stimuli. Downstream modulation of the MNS on the SMA and primary sensorimotor areas [34, 35] in response to MVF and delayed MVF might be essential for promoting motor network recovery during MT training for patients with paretic limbs. For patients with severe hand paresis, the ability to generate twice the activation in central cortical areas with a single movement using the delayed MVF in our MT system might be more efficient and superior than that achieved with the conventional MT setup.

Since the non-impaired hemisphere also has an ipsilateral control pathway to the impaired limb [36, 37], recruitment of the ipsilateral motor pathways during movement of the active hand has been considered by a recent review article as part of the MVF effects on motor recovery [6]. Since the measured cortical areas were reactivated when the active hand stopped moving in the P2 phase of the delayed-MVF condition (Fig. 3c and Table 2), this suggests that the recruitment of ipsilateral motor pathways might have played less of a role in the MVF effects on our healthy subjects. In addition, MVF is thought to evoke cognitive and perceptual conflicts in subjects with intact somatosensory pathways, especially when using unimanual movements [6]. These conflicts might largely activate the cortical attention network related to the unseen (paretic) hand in both healthy individuals [38] and patients with stroke [20, 39]. It is believed that increased attention toward the paralyzed limb is responsible for reconnecting the limb to the lesioned brain [11] and may help to disestablish the learned non-use state of the brain [6, 40]. Therefore, our finding of instant- and delayed-MVF effects on cortical activation might be explained in part from the attention perspective [6].

Moreover, it is interesting to note that the cortical responses (in terms of ERD waveforms and areas) during the P1 phase of the delayed-MVF condition were different from those in the no-MVF condition, but similar to those in the MVF condition (Fig. 3 and Table 2). The higher cortical activation observed during the P1 phase of the delayed-MVF condition compared to the no-MVF condition implies that the visual stimulus of the still hand might help to elevate cortical activity elicited from the button press. This may be because the image of the button plus the hand provides more dynamic information about the action of the button press than the image of the button alone. A previous study has found that merely viewing still pictures of hand gestures that imply action can significantly increase cortical activity [41]. Our results of significantly higher ERD areas during the P0 phase in the MVF and delayed-MVF conditions compared to the no-MVF condition (Table 2) may also support this viewpoint. Furthermore, cortical activation related to cognitive processes for the upcoming MVF should be considered. Given the action and perception experience of the movement in the delayed-MVF condition, the larger mu power decrease in this phase might be related to the anticipatory attention [42, 43]. Nevertheless, these speculations need to be studied further in the future by randomly arranging the regular MVF and delayed MVF in a single test condition.

Here, although mu ERD was found to be useful for analysing the temporal pattern of cortical activation in response to MVF stimuli, several methodological concerns should be addressed. First, we used self-paced movement rather than externally paced movement with a cued stimulus (auditory or visual) to simplify the stimulus content during the test conditions. It is believed that both types of movement involve similar brain structures, but that more complex processes are involved in externally paced movement including stimulus processing and expectancy [44]. Second, the low mu-rhythm presentation rate of our subject population (18 out of 28) may be due to the testing environment. Compared to previous studies [45, 46] that used dark environments and a semi-reclined position to record the mu-rhythm ERD, our testing environment was quite different. In our study, a bright environment was necessary for our test conditions, as the subjects needed to be able to see the MVF clearly on the LCD monitor. We also had the subjects sit in an upright position when performing the button presses and observing the MVF stimulus, which might have made the subjects less relaxed. Although the testing environment in our study was different from the environments in previous studies, ERD was still sensitive enough to detect the effects of the press movement and MVF on cortical areas. Moreover, the results we found using the overall and phasic ERD areas suggest that not only the contralateral sensorimotor area (detected by the C4 channel), but also the ipsilateral sensorimotor area (C3) and SMA (Cz) have similar and equal responses to MVF (Figs. 3 and 4). Our findings related to the no-MVF condition were generally in agreement with previous studies focused on mu suppression over central cortical areas during unimanual movement, which showed that mu rhythm desynchronization appears in the SMA and bilateral sensorimotor areas [23, 47, 48]. For the MVF conditions, previous studies using the lateralized readiness potential (LRP) have shown lateralized activation in the sensorimotor area of the contralateral hemisphere in response to the MVF in MT [49, 50]. Our lateralization analysis using the LI parameter (Table 3) also showed that the cortical responses to our MVF stimulus were more lateralized to the mirror (right) cortex compared to the cortical responses to the no-MVF condition, although the lower LI and larger variation indicate that inconsistent and imperfect lateralization was present. Coupling among all of the measured cortical areas in response to our MVF may need to be studied further in the future (especially for patients with stroke) to realize the clinical implications of our MT system. In summary, ERD can be used to determine the time-course changes of the effects of MT on the brain, but may not be sensitive enough to observe the lateralization of these effects.

## Conclusion

In this study, we successfully evolved traditional MT training to a form of digital MT with several features that help us better understand the effects of MVF on cortical activity. The temporal patterns of the cortical responses to delayed MVF have a number of important implications for future research and practice. In the future, this novel MT system could be used for bimanual coordination training in patients with stroke in both the symmetric and asymmetric modes. We hope that this new system will help promote and expand the use of MT for advanced movement training such as alternating and reciprocal bimanual training.

## Additional file

Additional file 1:
**The video demonstrates the system interface of our mirror therapy system and shows how we calibrate our camera before we use it.** Furthermore, we demonstrate the potential uses of our system for two different bimanual coordination training modes, i.e., symmetric and asymmetric modes with regular MVF (no delay) and 2-s delayed MVF, respectively.
